# Epidemiology and Burden of Gallbladder and Biliary Diseases and the Socioeconomic Factors in the Region of Middle East and North Africa, 1990–2021: Estimates From the Global Burden of Disease 2021 Study

**DOI:** 10.1002/hsr2.71829

**Published:** 2026-02-22

**Authors:** Zahra Isari, Melika Azizi Ghiasabadi, Mohammad Amin Gharihe, Amir Mashayekhi, Ali Khashaveh, Esmaeil Dabiri, Saeed Bahrampour, Arman Farsi, Kamiar Izadpanah, Hossein Pourghadamyari, Hamid Sharifi, Omid Eslami, Seyed Aria Nejadghaderi

**Affiliations:** ^1^ Student Research Committee, Razi Faculty of Nursing and Midwifery Kerman University of Medical Sciences Kerman Iran; ^2^ Student Research Committee, Afzalipour Faculty of Medicine Kerman University of Medical Sciences Kerman Iran; ^3^ Applied Cellular and Molecular Research Center Kerman University of Medical Sciences Kerman Iran; ^4^ Department of Clinical Biochemistry, Afzalipour Faculty of Medicine Kerman University of Medical Sciences Kerman Iran; ^5^ HIV/STI Surveillance Research Center, and WHO Collaborating Center for HIV Surveillance, Institute for Futures Studies in Health Kerman University of Medical Sciences Kerman Iran; ^6^ Student Research Committee Kerman University of Medical Sciences Kerman Iran; ^7^ Institute for Global Health Sciences University of California, San Francisco San Francisco CA USA; ^8^ Gastroenterology and Hepatology Research Center, Institute of Basic and Clinical Physiology Sciences Kerman University of Medical Sciences Kerman Iran; ^9^ Knowledge Hub for Migrant and Refugee Health, Institute for Futures Studies in Health Kerman University of Medical Sciences Kerman Iran

**Keywords:** burden of disease, disability adjusted life years, gallbladder diseases

## Abstract

**Background and Aims:**

As common disorders affecting the digestive system, gallbladder and biliary diseases present a significant health issue. This research aimed to assess the burden of these conditions in the Middle East and North Africa (MENA) region over the period of 1990 to 2021. Furthermore, we investigated the epidemiological characteristics of these diseases in relation to age, sex, and the Socio‐demographic Index (SDI).

**Methods:**

Drawing from the Global Burden of Disease (GBD) study 2021, we extracted data on the prevalence, incidence, and disability‐adjusted life years (DALYs) linked with gallbladder and biliary disease for 21 countries in the MENA region from 1990 to 2021. The results were presented as both absolute numbers and rates per 100,000 people, accompanied by their corresponding 95% uncertainty intervals (UIs). The association between disease burden and SDI was evaluated using smoothing spline models.

**Results:**

In 2021, the MENA region documented 4.8 million new cases of gallbladder and biliary diseases, with an age‐standardized incidence rate of 808.9 per 100,000 population, which represents a 5.0% reduction since 1990. The prevalence increased to 15.4 million cases, with an age‐standardized rate of 2807.5 per 100,000, marking a 7.9% decrease. The overall disease burden, quantified in DALYs, reached 437.1 thousand, with an age‐standardized rate of 85.7 per 100,000, signifying a 16.6% decline. Afghanistan estimated the highest DALY rates, while Oman had the lowest. A more pronounced burden was observed in females across all age brackets. An inverse correlation was identified between disease burden and SDI.

**Conclusion:**

Gallbladder and biliary diseases continue to be a public health challenge, particularly affecting women, the elderly, and nations with lower SDI levels. It is imperative for each country to develop healthcare systems and preventive measures that are tailored to its specific context.

## 1 Introduction

Disorders of the gallbladder and biliary system are among the most prevalent digestive tract ailments. This spectrum of conditions encompasses gallbladder issues like gallstones and cholecystitis, as well as biliary tree problems such as choledocholithiasis [1]. Key risk factors associated with the development of these diseases include female sex, advancing age, pregnancy, obesity, and a sedentary lifestyle [2, 3]. These diseases can lead to various complications, including cholecystitis, gallbladder perforation, pericholecystic abscess, emphysematous cholecystitis, pancreatitis, acute cholangitis, and gallbladder cancer [4]. Gallstone disease is the most common and costly condition among digestive diseases, with an estimated prevalence of 10%–20% in the Western world [5].

Globally, in 2021, the age‐standardized incidence and mortality rates for gallbladder and biliary disease were reported as 865.4 and 1.6 per 100,000 population, respectively. The most substantial incidence rates were seen in countries with a high Socio‐demographic Index (SDI). Females experienced a greater number of incident cases and mortality rates than males globally. Globally, females experienced more incident cases and higher mortality rates than males. Additionally, the number of incident cases among females increased with age, reaching its peak in the 50–54 age group [2].

Previous analyses utilizing the Global Burden of Disease (GBD) 2019 data have investigated the epidemiology of these diseases at a global level [6, 7]. Recently, the worldwide burden of these diseases has been analyzed using age‐period‐cohort methods [2, 8]. However, these studies did not specifically address the epidemiological patterns or the age and sex distributions of gallbladder and biliary diseases in the Middle East and North Africa (MENA) region. Given that MENA is a transcontinental area with distinct cultural, societal, religious, economic, and ethnic diversities [9], these factors may impact the burden of these diseases. The region is undergoing a rapid nutritional transition, with obesity rates among the highest globally and high diabetes prevalence in several nations, both risk factors for gallstone formation. Additionally, high consanguinity rates may amplify genetic predispositions to cholelithiasis, while elevated chronic hepatitis B/C infections and parasitic diseases like echinococcosis can lead to secondary biliary complications not highlighted in global aggregates [10]. While the GBD study provides regional estimates, aggregated MENA data do not capture within‐region variations related to distinct healthcare systems, socioeconomic transitions, and demographic structures. Therefore, a sub‐regional analysis is crucial to understand country‐specific trends and inform region‐tailored public health policies.

By exploring within‐region heterogeneity and the influence of SDI on disease burden, this analysis provides evidence to guide health‐system priorities and preventive strategies tailored to national contexts, such as addressing surgical access gaps in conflict zones (e.g., Yemen, Syria) where untreated gallstones lead to higher complication rates, or promoting metabolic risk screening in countries with high prevalence of obesity. This study aimed to evaluate the burden of gallbladder and biliary diseases in the MENA region from 1990 to 2021, while also exploring epidemiological trends according to age, sex, and SDI.

## 2 Methods

### 2.1 Overview

This study utilized data from the GBD 2021, a comprehensive project led by the Institute for Health Metrics and Evaluation at the University of Washington, which aims to quantify health loss across the globe and accessed at https://vizhub.healthdata.org/gbd-results/ on 12 September 2024. The present study was approved by the ethics committee of Kerman University of Medical Sciences (IR. KMU. REC.1404.025).

The GBD 2021 study provides estimates for 371 diseases and injuries and 88 risk factors across 204 countries and territories, which are grouped into 21 regions and seven super‐regions. Key metrics estimated in GBD 2021 include incidence, mortality, prevalence, years of life lost (YLLs), years lived with disability (YLDs), and disability‐adjusted life years (DALYs). This study focuses on presenting the burden of gallbladder and biliary diseases from 1990 to 2021 for the 21 countries that constitute the MENA region. The MENA region is one of the 21 GBD regions and is also classified as one of the seven GBD super‐regions. Also, country inclusion followed the GBD regional classification used in GBD 2021, which defines the MENA region as comprising 21 countries.

### 2.2 Case Definition

In the context of the GBD study, gallbladder and biliary diseases encompass conditions such as cholecystitis, gallstones, cholangitis, and other related disorders of the gallbladder and biliary tract. Gallstones, which are solid crystalline structures formed from cholesterol, phospholipids, bile pigments, or calcium salts, can lead to abdominal pain. Cholecystitis is defined as inflammation of the gallbladder, and cholangitis is the inflammation of the bile ducts, frequently caused by blockages from gallstones. Symptoms of these conditions can include severe abdominal pain, nausea, vomiting, fever, and jaundice. It is important to note that cancers of the gallbladder and biliary tract are classified separately and are not included in this disease category [11]. Disease classification is based on the International Classification of Diseases (ICD), using versions 9 and 10. For GBD 2021, the ICD‐9 codes for these diseases span from 574 to 576.9, and the ICD‐10 codes range from K80 to K83.9. Specifically, K80 corresponds to cholelithiasis, K81 to cholecystitis, K82 to other gallbladder diseases, and K83 to other biliary tract diseases. This analysis excluded all malignant neoplasms (ICD‐10: C23–C24). Only non‐malignant conditions (K80–K83.9) were included, consistent with GBD 2021 definitions [12].

### 2.3 Data Sources

Mortality associated with gallbladder and biliary illnesses was estimated by the GBD 2021 framework using vital registry data from the Cause of Death database. No individual‐level data were used. A systematic review of data for each location and year was performed to detect outliers. Data points that contradicted established age or time trends were excluded from the analysis. Furthermore, instances where the combination of redistributing garbage codes and noise reduction led to irrational cause fractions, especially with small sample sizes, were also omitted [12]. All statistical modeling was conducted by the GBD research team. The present authors performed secondary analyses, including calculation of percentage changes and visualization using R software (version 4.4.2). Data were analyzed in R version 4.4.2 using the following packages: ggplot2, dplyr, and splines. All descriptive and correlation analyses followed IHME's standardized analytical framework.

### 2.4 Disease Modeling and Statistical Analysis

All primary disease modeling and estimation for gallbladder and biliary diseases were performed by the GBD 2021 collaborators at the Institute for Health Metrics and Evaluation, using standardized methodologies detailed in the GBD 2021 capstone papers [11, 12]. To estimate mortality rates by location, sex, and age, the cause of death ensemble model (CODEm) was employed by the GBD team. Separate CODEm models were constructed for male and female mortality, covering age ranges from 2 years to 95+ years. The process began by combining global and data‐rich models to generate unadjusted results. Subsequently, these results were adjusted using the CoDCorrect algorithm. The mortality modeling for gallbladder and biliary diseases incorporated several covariates, including mean body mass index, the healthcare access and quality index, and the SDI [12].

YLLs were calculated in the GBD framework by subtracting the age of death from the GBD reference life table to determine the remaining life expectancy for each death. The death rates in each age group were then multiplied by the group's remaining life expectancy to estimate YLLs [12]. The severity distribution of gallbladder and biliary diseases was divided into three levels based on symptoms and their effect on quality of life, each with a specific disability weight (DW). YLDs were calculated by multiplying the prevalence rates for each severity level by their corresponding DWs, with DWs derived from population surveys and adjusted for comorbidity using simulation methods.

In this study, the authors performed secondary analyses on the extracted GBD data, including computation of age‐standardized rates and counts, together with their 95% Uncertainty Intervals (UIs). Smoothing spline models were utilized to investigate the association between the SDI and the burden of these diseases [13]. No inferential hypothesis testing was conducted; instead, 95% UIs were reported to convey precision of estimates. Data visualization and statistical analyses were conducted using the R software (version 4.4.2; R Foundation for Statistical Computing, Vienna, Austria) with the following packages: ggplot2, dplyr, and splines. The SDI is a composite indicator based on per capita income, the mean number of years of education for those aged 15 and older, and the fertility rate for women younger than 25. It is scaled from zero to one, representing a spectrum from the lowest to the highest level of development [12]. SDI was selected over alternative metrics like the Human Development Index because it is embedded within the GBD framework, avoids circularity by excluding health components, and provides a standardized measure tailored for correlating socioeconomic development with health burdens.

## 3 Results

### 3.1 Regional Level

In 2021, the MENA region estimated 4.8 million (95% UI: 4.0, 5.8 million) new incident cases of gallbladder and biliary diseases, corresponding to an age‐standardized incidence rate of 808.9 per 100,000 population (95% UI: 685.8, 968.0). This incidence rate represented a significant decrease from 1990 (−5.0% [−7.5, –2.3]). In the same year, 15.4 million people were living with gallbladder and biliary diseases (95% UI: 12.9, 18.6 million). The age‐standardized prevalence rate for 2021 was 2807.5 per 100,000 population (95% UI: 2370.2, 3359.4), which also reflected a decrease since 1990 (−7.9% [−10.6, –4.7]). The total DALYs attributed to these diseases were 437.1 thousand (322.2, 603.4) in 2021, with a corresponding rate of 85.7 (64.7, 115.9) per 100,000 population. Although age‐standardized rates have declined significantly since 1990 (−16.6% [−26.8, –5.4]), the absolute numbers of incident and prevalent cases increased, reflecting population growth and aging (Table 1).

**Table 1:** Prevalent cases, incident cases, and DALYs, along with their age‐standardized rates, due to gallbladder and biliary diseases in 2021 for both sexes and the percentage change in rates per 100,000 population from 1990 to 2021 in the Middle East and North Africa region.

Location	Incidence (95% UI)			Prevalence (95% UI)			DALYs (95% UI)		
	Counts (2021)	Rate (2021)	Pcs in rate 1990–2021	Counts (2021)	Rate (2021)	Pcs in rate 1990–2021	Counts (2021)	Rate (2021)	Pcs in rate 1990–2021
Middle East and North Africa	4814453.7 (4039683.5, 5821331.1)	808.9 (685.8, 968.0)	−5.0 (−7.5, −2.3)	15409320.3 (12897681.7, 18589707.9)	2807.5 (2370.2, 3359.4)	−7.9 (−10.6, −4.7)	437067.4 (322226.1, 603377.2)	85.7 (64.7, 115.9)	−16.6 (−26.8, −5.4)
Afghanistan	192253.2 (158214.0, 230222.3)	1033.5 (859.5, 1223.0)	−3.4 (−10.6, 5.3)	595141.3 (502027.8, 703910.7)	3783.0 (3165.2, 4499.9)	1.3 (−6.0, 10.1)	17562.0 (11862.9, 23800.8)	126.2 (86.7, 171.6)	−0.1 (−18.5, 18.7)
Algeria	342124.5 (287855.4, 412715.1)	782.9 (661.1, 934.9)	−6.5 (−14.2, 0.8)	1105347.2 (934109.6, 1321864.2)	2681.4 (2261.9, 3203.4)	−9.5 (−16.5, −2.3)	29385.9 (20661.8, 42255.8)	76.1 (54.7, 108.0)	−9.5 (−23.8, 7.7)
Bahrain	11574.3 (9627.6, 14273.4)	716.5 (605.6, 858.1)	−20.4 (−25.8, −14.8)	34754.7 (29020.0, 42935.5)	2527.7 (2133.2, 3056.1)	−24.8 (−30.0, −19.4)	1053.0 (747.1, 1487.4)	102.7 (77.0, 139.4)	−32.4 (−45.3, −10.5)
Egypt	730904.6 (608413.4, 896367.9)	814.6 (682.0, 995.1)	−1.3 (−8.6, 6.1)	2332498.8 (1950793.9, 2841435.3)	2869.3 (2406.5, 3500.1)	−2.8 (−9.5, 4.2)	69354.8 (48652.6, 95559.6)	96.7 (68.1, 132.4)	−30.3 (−51.0, 3.3)
Iran	701618.6 (586944.6, 839462.4)	753.8 (640.6, 889.9)	−0.3 (−3.1, 2.8)	2260195.6 (1896994.7, 2696031.8)	2580.0 (2172.1, 3061.2)	−3.9 (−7.0, −0.5)	58902.8 (41156.6, 82780.2)	70.0 (50.1, 96.8)	−4.9 (−14.1, 6.6)
Iraq	256920.3 (210582.0, 313775.3)	718.1 (594.3, 876.0)	−10.1 (−16.1, −2.5)	795899.7 (652286.6, 973132.2)	2470.4 (2062.6, 3000.3)	−12.9 (−18.8, −5.7)	19646.9 (13399.7, 28061.6)	63.8 (44.4, 90.2)	−17.4 (−27.8, −5.3)
Jordan	116727.6 (99615.1, 137159.5)	1033.8 (890.9, 1200.7)	1.7 (−5.2, 9.4)	355214.9 (302710.6, 418068.9)	3495.7 (2991.0, 4059.6)	−4.5 (−11.4, 2.8)	9252.5 (6492.2, 12994.0)	99.1 (70.2, 136.3)	−20.9 (−33.6, −4.1)
Kuwait	40855.6 (33853.9, 50604.3)	749.8 (627.3, 911.2)	8.9 (−1.0, 19.1)	113193.6 (93912.6, 137530.5)	2412.4 (2014.8, 2905.6)	−0.6 (−9.5, 7.9)	2809.3 (1951.5, 4017.5)	65.5 (46.9, 90.7)	−7.3 (−16.3, 0.6)
Lebanon	46855.6 (39265.5, 56288.9)	784.7 (659.1, 937.6)	−0.4 (−7.0, 7.3)	153830.1 (129579.7, 185516.7)	2585.3 (2164.1, 3133.1)	−5.9 (−12.0, 1.0)	5320.4 (4111.9, 6962.8)	86.9 (67.0, 114.7)	−23.3 (−41.2, 7.0)
Libya	57773.1 (47629.8, 70409.0)	778.8 (655.5, 931.8)	2.3 (−4.8, 9.8)	181182.9 (150361.0, 220687.2)	2701.7 (2263.3, 3256.8)	−0.9 (−8.0, 6.5)	5340.2 (3698.2, 7559.6)	87.2 (60.2, 123.9)	7.0 (−13.6, 37.9)
Morocco	321817.0 (263219.2, 392057.6)	834.5 (686.2, 1008.4)	−2.8 (−10.1, 5.4)	1099002.0 (900351.9, 1337348.4)	2957.6 (2443.1, 3572.7)	−3.3 (−10.8, 4.9)	30881.5 (21097.6, 42643.7)	86.7 (59.7, 119.2)	−1.2 (−13.4, 13.8)
Oman	27453.0 (22442.2, 34075.5)	611.2 (510.8, 742.0)	−6.9 (−14.2, 0.5)	78688.2 (65048.4, 96931.7)	2114.7 (1769.2, 2544.2)	−12.5 (−19.0, ‐5.7)	1986.0 (1338.6, 2935.5)	59.7 (42.6, 83.7)	−21.9 (−35.5, −6.2)
Palestine	31070.5 (25717.2, 37808.3)	794.6 (674.0, 954.5)	−8.6 (−15.9, −0.9)	96021.2 (80253.3, 116388.6)	2778.6 (2340.8, 3326.1)	−10.4 (−16.5, −3.0)	2560.6 (1805.6, 3631.1)	81.8 (60.6, 112.1)	−29.2 (−45.2, −12.4)
Qatar	19390.2 (15685.6, 24317.1)	647.7 (541.8, 770.5)	3.4 (−3.5, 12.7)	52241.5 (42013.5, 65223.4)	2252.1 (1870.8, 2683.0)	−3.5 (−10.0, 4.6)	1260.3 (809.9, 1911.5)	64.3 (45.8, 91.5)	−22.3 (−38.6, 0.6)
Saudi Arabia	324121.9 (265714.6, 407691.1)	820.8 (686.5, 1004.4)	−6.6 (−13.7, 0.3)	928521.3 (767505.8, 1152271.5)	2831.1 (2358.6, 3395.1)	−12.7 (−19.0, −6.0)	29555.5 (21384.6, 41078.3)	108.5 (80.3, 146.5)	−20.1 (−38.0, 2.7)
Sudan	260238.0 (217978.7, 314749.6)	820.0 (693.9, 997.1)	−2.2 (−10.9, 4.6)	816084.4 (690693.0, 974478.2)	2924.5 (2464.5, 3533.8)	−2.3 (−10.1, 5.1)	23273.3 (16205.8, 32489.4)	90.7 (64.1, 126.7)	2.4 (−14.5, 25.2)
Syrian Arab Republic	112863.4 (92586.6, 137504.0)	769.0 (635.6, 928.0)	−2.9 (−11.3, 4.7)	368748.6 (303240.7, 453573.4)	2558.4 (2129.6, 3112.4)	−8.4 (−15.5, −1.2)	9192.9 (6345.3, 13230.0)	66.4 (47.0, 93.8)	−12.3 (−23.1, −1.0)
Tunisia	86958.8 (72652.7, 105325.1)	642.5 (539.1, 770.6)	−0.1 (−6.7, 7.4)	293170.5 (245515.0, 355462.0)	2181.3 (1839.4, 2624.8)	−4.6 (−10.9, 1.9)	8354.3 (5797.6, 12017.3)	63.9 (45.3, 91.8)	−3.9 (−18.9, 17.7)
Turkey	848474.4 (702436.1, 1032588.7)	887.7 (738.3, 1076.2)	−10.2 (−17.6, −1.9)	2862149.3 (2360068.9, 3470606.2)	3014.9 (2500.3, 3639.6)	−15.2 (−22.7, −7.2)	87877.0 (64204.1, 121471.0)	95.1 (70.3, 130.3)	−23.6 (−37.1, −7.6)
United Arab Emirates	73718.0 (58565.5, 93111.4)	604.9 (508.3, 713.9)	−8.5 (−15.4, −1.8)	219425.2 (175163.7, 275512.5)	2181.4 (1830.6, 2572.9)	−13.5 (−20.0, −6.7)	5324.3 (3526.2, 7727.0)	73.8 (54.0, 100.2)	−17.8 (−37.8, 20.1)
Yemen	206250.6 (171621.4, 250035.7)	886.4 (751.7, 1057.6)	0.5 (−8.2, 9.1)	653637.0 (548786.1, 789139.7)	3224.9 (2730.2, 3831.2)	1.1 (−6.8, 10.3)	17766.4 (12521.0, 24329.4)	96.8 (69.8, 129.5)	5.2 (−10.8, 22.5)

Abbreviations: DALY, disability‐adjusted life year; Pcs,percent changes; U, uncertainty interval.

### 3.2 Country Level

**Figure 1:**
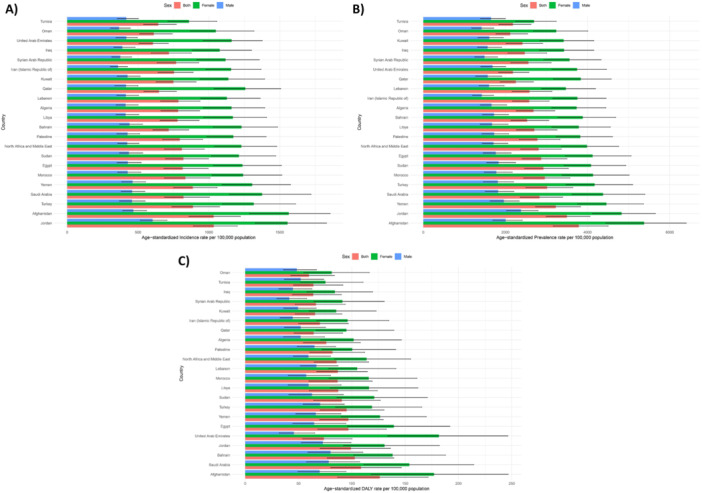
Age‐standardized incidence (A), prevalence (B), and DALYs (C) for gallbladder and biliary diseases (per 100,000 population) in the Middle East and North Africa region in 2021, by sex and country. DALYs, disability‐adjusted‐life‐years. Bars represent rates per 100,000 population (x‐axis) for each country (y‐axis), with blue bars indicating males, green bars indicating females, red bars indicating both, and error bars showing 95% uncertainty intervals. Axes units: x‐axis (rates per 100,000 population), y‐axis (countries ordered by descending rates).

Within the region, there was significant variation in the age‐standardized incidence rate of gallbladder and biliary diseases, which in 2021 spanned from 604.9 to 1033.8 per 100,000 population. The countries with the highest age‐standardized incidence rates were Jordan (1033.8 [890.9, 1200.7]), Afghanistan (1033.5 [859.5, 1223.0]), and Turkey (887.7 [738.3, 1076.2]). Conversely, the United Arab Emirates (604.9 [508.3, 713.9]), Oman (611.2 [510.8, 742.0]), and Tunisia (642.5 [539.1, 770.6]) documented the lowest age‐standardized incidence rates in 2021 (Table 1 and Figure 1A).

The age‐standardized point prevalence of these diseases in the region ranged from 2114.7 to 3783.0 cases per 100,000. In 2021, the highest age‐standardized point prevalence in MENA was observed in Afghanistan (3783.0 [3165.2, 4499.9]), Jordan (3495.7 [2991.0, 4059.6]), and Yemen (3224.9 [2730.2, 3831.2]), while the lowest rates were seen in Oman (2114.7 [1769.2, 2544.2]), Tunisia (2181.3 [1839.4, 2624.8]), and the United Arab Emirates (2181.4 [1830.6, 2572.9]) (Table 1 and Figure 1B).

The age‐standardized rate of DALYs for gallbladder and biliary diseases in 2021 varied from 59.7 to 126.2 cases per 100,000 in the MENA region. Afghanistan (126.2 [86.7, 171.6]), Saudi Arabia (108.5 [80.3, 146.5]), and Bahrain (102.7 [77.0, 139.4]) documented the highest age‐standardized DALYs rates, whereas Oman (59.7 [42.6, 83.7]), Iraq (63.8 [44.4, 90.2]), and Tunisia (63.9 [45.3, 91.8]) had the lowest rates (Table 1 and Figure 1C).

A decline in the age‐standardized incidence rate of gallbladder and biliary illnesses was observed in all MENA nations, with the exception of Libya, Yemen, Qatar, Kuwait, and Jordan (Figure S1). A similar reduction was noted in the age‐standardized point prevalence, except for Afghanistan and Yemen (Figure S2). Furthermore, age‐standardized DALY rates decreased in most countries, with the exceptions of Sudan, Libya, and Yemen (Figure S3).

### 3.3 Burden of Gallbladder and Biliary Diseases by Age and Sex

**Figure 2:**
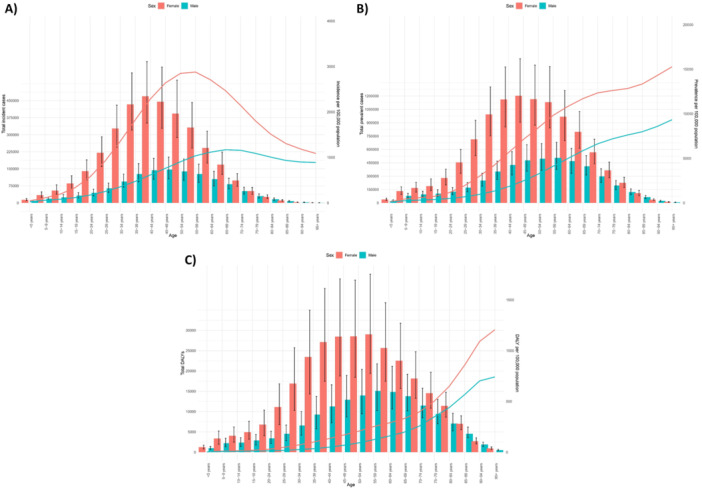
Total incident cases and incidence rate (A), total prevalent cases and point prevalence (B), and total DALYs and DALY rate (C) of gallbladder and biliary diseases (per 100,000 population) in the Middle East and North Africa region, by age and sex in 2021. DALYs, disability‐adjusted‐life‐years. Lines represent rates for males (blue) and females (red). Bars represent numbers for males (blue) and females (red). Error bars showing 95% uncertainty intervals for numbers.

For both sexes, the total number of incident cases of gallbladder and biliary diseases began to rise in children under 5 years old, peaking before declining in older age groups. The peak age range for the total number of incident cases was 40–44 years for females and 45–49 years for males. Similarly, incidence rates per 100,000 population for both sexes also increased from the under‐5 age group, reaching their highest point in the 55–59 age group for females and the 65–69 age group for males, before decreasing with older age (Figure 2, 2A). The total number of prevalent cases also increased from the under‐five age group and then declined after peaking in the 45–49 age range for females and the 55–59 range for males. In contrast, the prevalence rate increased steadily with advancing age (Figure 2B). Moreover, the total number of DALYs increased until it peaked in the 55–59 age group, after which it fell. The DALY rate, however, showed a continuous upward trend, initially rising gently and then more steeply (Figure 2C). In every age group, females had substantially higher numbers and rates of incidence, prevalence, and DALYs.

### 3.4 SDI Association

SDI was selected instead of HDI because it is an internally consistent index within the GBD framework, combining income, education, and fertility rate to better capture demographic and economic influences on disease burden.

**Figure 3:**
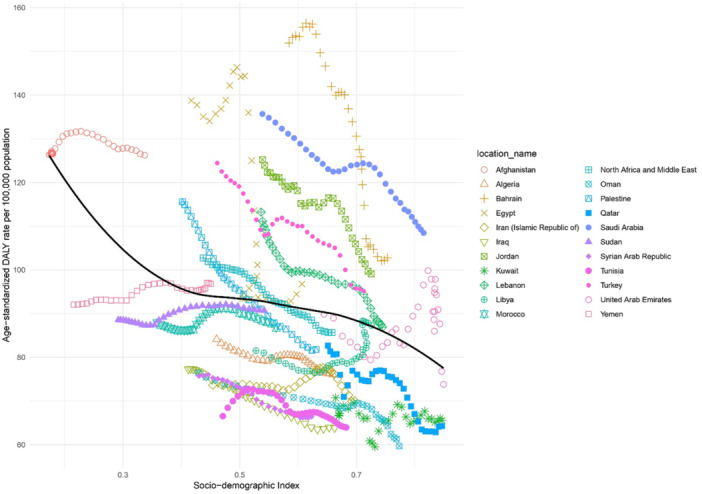
Age‐standardized DALY rates of gallbladder and biliary diseases for the 21 countries, by SDI. The black line represents expected DALY rates based on the relationship between SDI and disease burden. Each point on the plot indicates the observed age‐standardized DALY rate for a specific country in 2021. DALYs, disability‐adjusted‐life‐years. SDI, Socio‐demographic Index. Points are colored by location. Axes units: y‐axis (Age‐standardized DALY rates per 100,000 population), x‐axis (SDI score, 0–1).

An inverse relationship was observed between the SDI level and age‐standardized DALY rates for biliary and gallbladder diseases, with significant variations across the region. Bahrain, Turkey, Jordan, and Saudi Arabia showed substantially higher rates than would be expected based on their SDI level for the MENA region. In contrast, Tunisia, Algeria, Iraq, Oman, Sudan, Qatar, and Iran displayed lower age‐standardized DALY rates than expected for their SDI levels in MENA (Figure 3).

## 4 Discussion

We found that in 2021, there was still a significant burden from gallbladder and biliary diseases, despite a decreasing trend over the 1990–2021 period. The burden was higher in females. Additionally, we found that improved socioeconomic status, assessed by SDI, reduced the burden of this condition in MENA. Although our findings are consistent with global patterns reported by He et al. [2], our study extends their work by providing a focused sub‐regional analysis with country‐specific SDI correlations and updated 2021 data.

Our results showed that MENA has experienced a decline in age‐standardized incidence, prevalence, and DALY rates for gallbladder and biliary diseases over the past three decades, which aligns with the global rates regarding the burden of these diseases. In this context, the estimated annual percentage changes in incidence and DALY rates were *−*0.32% and −0.69%, respectively [2]. However, the observed reductions in age‐standardized incidence rate were −5.0%, in prevalence rate ‐7.9%, and in DALY rate −16.6% since 1990. These differences between global rates and the MENA values may stem from improved healthcare access (including disease management and prevention measures) [14], reduced infections, and public health efforts that promote healthier diets [15] as well as genetic factors [16, 17]. Notably, the rapid nutritional transition in MENA, characterized by increased consumption of high‐fat, high‐carbohydrate processed foods, fast food, and red meat has contributed to rising obesity rates, a factor for gallstone formation, though targeted interventions in some nations have reduced this [18, 19]. Meanwhile, global rates continue to be influenced by rising obesity and metabolic risks [20], which targeted infection control and public health efforts in MENA have declined. Compared with other GBD regions, the MENA's decline in age‐standardized DALYs (−16.6%) was steeper than that of South Asia (−11.4%) but slower than that of High‐Income regions (−22.5%), reflecting intermediate progress in healthcare system strengthening [2].

The variation in the burden of these conditions across different countries in MENA was considerable. Countries such as Jordan, Afghanistan, and Turkey had higher incidence rates, while Oman, Tunisia, and the United Arab Emirates had lower rates. This variation likely reflects differences in healthcare systems, access to medical care, regional lifestyle factors like dietary modifications, and genetic predispositions [9, 17]. In this regard, the high DALY rates in Afghanistan might be exacerbated by ongoing conflict limiting access to diagnostic ultrasound and safe cholecystectomy, leading to higher rates of complications like biliary perforation. In addition, high parasitic burdens, such as Ascaris lumbricoides infestation and self‐medication practices among women further increase untreated cases and mortality [21]. In contrast, the low rates in Oman can be due to policy‐driven investments in primary care infrastructure, including widespread point‐of‐care imaging and elective surgery programs, which have improved early detection and reduced complications. Genetic factors, such as familial predispositions observed in Pakistani populations and high consanguinity rates may increase risks in lower‐SDI countries, interacting with environmental stressors like hemolytic conditions in children [22]. Moreover, the significant reduction of 16.6% in disease burden compared to 1990, measured by DALYs, indicates progress in managing morbidity and mortality associated with gallbladder and biliary diseases in this region. Nevertheless, Afghanistan, Saudi Arabia, and Bahrain reported the highest DALY rates in 2021 within MENA, which may be linked to increasingly sedentary lifestyles, obesity, smoking, and secondhand smoke exposure [15, 18, 23]. The findings of our study show that females have higher incidence, prevalence, and DALYs rates than males in each age group, which is consistent with the study by He et al. that demonstrated that being female, being older, and having a high body mass index are significant risk factors for the global burden of gallbladder and biliary diseases from 1990 to 2021 [2]. Hormonal and metabolic differences, such as higher rates of obesity, pregnancy‐related changes, and contraceptive use, may contribute to earlier disease onset in women by reflecting an earlier increase in incidence peaks among females [18, 23].

In accordance with the global pattern of DALY rates of gallbladder and biliary diseases over the period 1990*–*2021 [2], DALY rates in MENA continued to increase as age grew, initially with a gentle slope and then sharper (after 65–69 years). This trend may be attributed to frequent medical check‐ups in the elderly, making diagnosing gallbladder conditions easier. To decrease the burden of disease, nations should prioritize the elderly through MENA‐tailored strategies, such as promoting traditional low‐fat Mediterranean dietary elements to counter the shift toward high‐salt, high‐fat Western diets; integrating moderate physical activity programs adapted to cultural norms; and enhancing primary care systems with affordable ultrasound screening and obesity prevention campaigns, as recommended by regional health bodies for MENA [24].

We found an inverse SDI‐DALY relationship that follows the global association [2]. This finding indicates a correlation between socioeconomic development and lower disease burden, without implying direct causality. In high‐SDI countries, the public health care system supports physical activity [20, 25] and healthier diets [26], and this can mitigate risk factors. In addition, Khanali and colleagues showed that the quality of care increased with SDI levels for gallbladder and biliary tract cancers [27]. This also explains how better care could lower the burden of diseases in areas with high SDI. It emphasizes the need for nations to make investments in healthcare systems in order to improve early diagnosis and surgical access [28].

To the best of our knowledge, this study is the first to present the most up‐to‐date data on the regional burden and epidemiology of biliary and gallbladder diseases in the MENA region, as well as how these conditions relate to socioeconomic factors. Nevertheless, there are three limitations. First, the absence of high‐quality data categorized by particular diseases or anatomical regions could potentially induce bias. Second, the GBD study used a different modeling technique to estimate the burden in nations where data were unavailable. This means that the results may be based on estimates rather than actual data, particularly in areas where high‐quality information is scarce. Third, racial and ethnic groups, as well as data specific to rural and urban areas, were not reported separately in this study. Additionally, since the study relied on secondary data, unmeasured confounding and potential misclassification errors cannot be ruled out. Country‐level differences in diagnostic capacity and data reporting completeness may also contribute to variability. Primary data quality and modeling strategies can be enhanced in future GBD iterations.

## 5 Conclusions

Over the past three decades, the burden of these diseases has decreased throughout MENA; yet, these conditions still present a public health concern, particularly for women and the elderly. Countries and regions with low SDI levels had noticeable disparities in the burden of these diseases. Tailored policy actions are required across MENA, including strengthening preventive nutrition programs, ensuring affordable imaging and surgical access, and integrating lifestyle interventions targeting women and older adults in lower‐SDI settings.

## Author Contributions

**Zahra Isari:** data curation, methodology, writing – original draft, writing – review and editing. **Melika Azizi Ghiasabadi:** data curation, methodology, writing – original draft, writing – review and editing. **Mohammad Amin Gharihe:** data curation, methodology, writing – original draft, writing – review and editing. **Amir Mashayekhi:** data curation, methodology, writing – original draft, writing – review and editing. **Ali Khashaveh:** writing – original draft, writing – review and editing. **Esmaeil Dabiri:** writing – original draft, writing – review and editing. **Saeed Bahrampour:** writing – original draft, writing – review and editing. **Arman Farsi:** writing – original draft, writing – review and editing. **Kamiar Izadpanah:** writing – original draft, writing – review and editing. **Hossein Pourghadamyari:** validation, writing – review and editing, writing – original draft, conceptualization, project administration, resources, supervision. **Hamid Sharifi:** supervision, writing – review and editing, writing – original draft, conceptualization, methodology, validation, project administration, resources. **Omid Eslami:** conceptualization, supervision, writing – original draft, writing – review and editing. **Seyed Aria Nejadghaderi:** methodology, conceptualization, writing – original draft, writing – review and editing, validation, supervision, project administration, resources, investigation. All authors have read and approved the final version of the manuscript. Seyed Aria Nejadghaderi and Omid Eslami had full access to all of the data in this study and take complete responsibility for the integrity of the data and the accuracy of the data analysis.

## Ethics Statement

The present study was approved by the ethics committee of Kerman University of Medical Sciences, Kerman, Iran (IR.KMU.REC.1404.025).

## Conflicts of Interest

The authors declare no conflicts of interest.

## Transparency Statement

The lead authors Omid Eslami and Seyed Aria Nejadghaderi affirm that this manuscript is an honest, accurate, and transparent account of the study being reported; that no important aspects of the study have been omitted; and that any discrepancies from the study as planned (and, if relevant, registered) have been explained.

## Supporting information


**Figure S1:** The percentage change in the age‐standardized incidence per 100,000 population of gallbladder and biliary diseases in the Middle East and North Africa region from 1990 to 2021, by sex and country. **Figure S2:** The percentage change in the age‐standardized prevalence per 100,000 population of gallbladder and biliary diseases in the Middle East and North Africa region from 1990 to 2021, by sex and country**. Figure S3:** The percentage change in the age‐standardized DALY per 100,000 population of gallbladder and biliary diseases in the Middle East and North Africa region from 1990 to 2021, by sex and country. DALY = disability‐adjusted‐life‐year.

## Data Availability

The authors confirm that all data supporting the findings are publicly available via the GBD Results Tool (https://vizhub.healthdata.org/gbd-results/). No additional data were generated.
