# Supplementary material for: Epidemiology and Burden of Gallbladder and Biliary Diseases and the Socioeconomic Factors in the Region of Middle East and North Africa, 1990–2021: Estimates From the Global Burden of Disease 2021 Study
Source: Health Sci Rep. 2026 Feb 22;9(2):e71829. doi: 10.1002/hsr2.71829 (PMC12928078; doi:10.1002/hsr2.71829)
Supplement: Supplementary file 1 — Figure S1: The percentage change in the age‐standardized incidence per 100,000 population of gallbladder and biliary diseases in the Middle East and North Africa region from 1990 to 2021, by sex and country. Figure S2: The percentage change in the age‐standardized prevalence per 100,000 population of gallbladder and biliary diseases in the Middle East and North Africa region from 1990 to 2021, by sex and country. Figure S3: The percentage change in the age‐standardized DALY per 100,000 population of gallbladder and biliary diseases in the Middle East and North Africa region from 1990 to 2021, by sex and country. DALY = disability‐adjusted‐life‐year. [file HSR2-9-e71829-s001.docx]

**
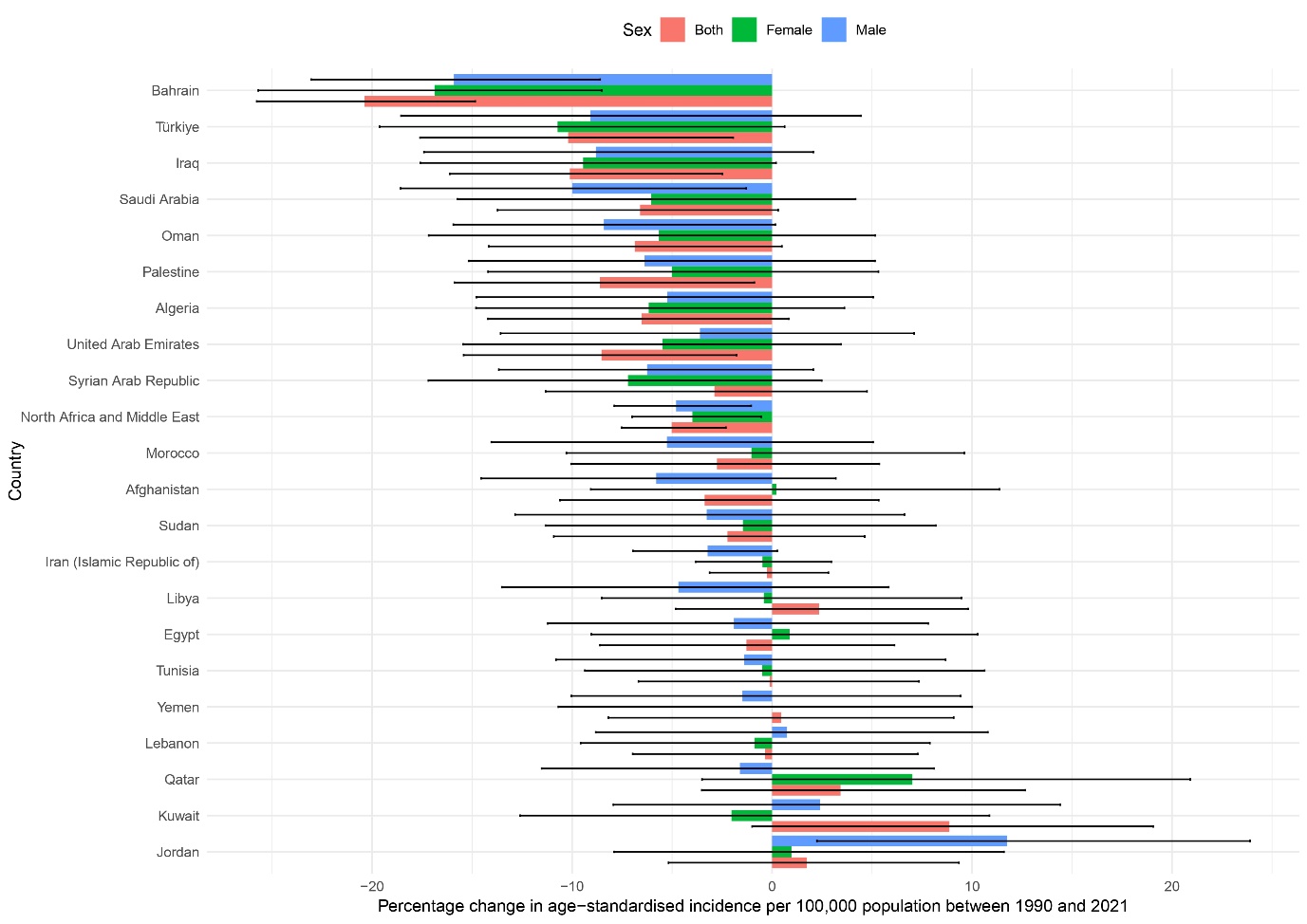
**

**Figure S1.** The percentage change in the age-standardized incidence per 100,000 population of gallbladder and biliary diseases in the Middle East and North Africa region from 1990 to 2021, by sex and country.

**
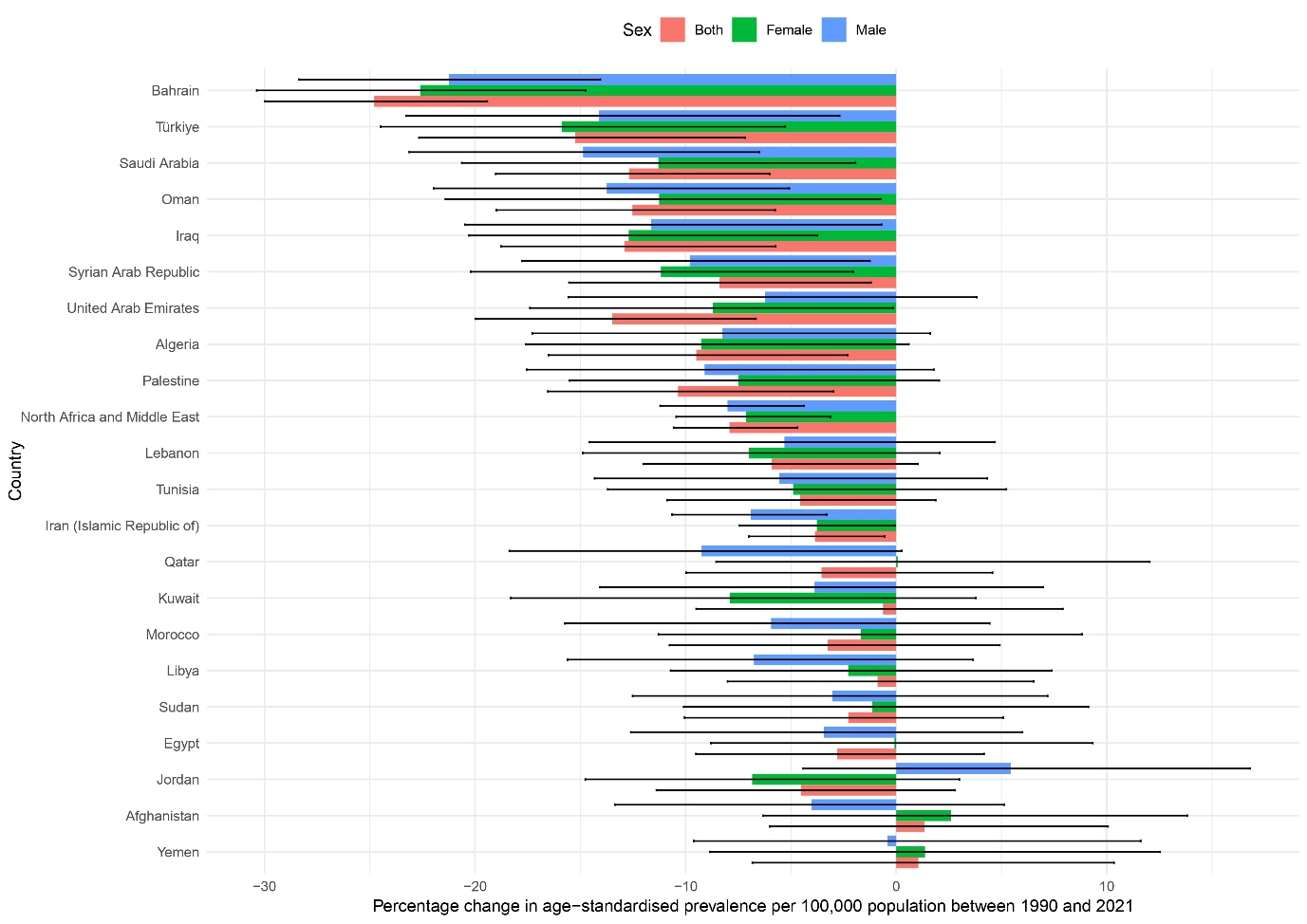
**

**Figure S2.** The percentage change in the age-standardized prevalence per 100,000 population of gallbladder and biliary diseases in the Middle East and North Africa region from 1990 to 2021, by sex and country**.**

**
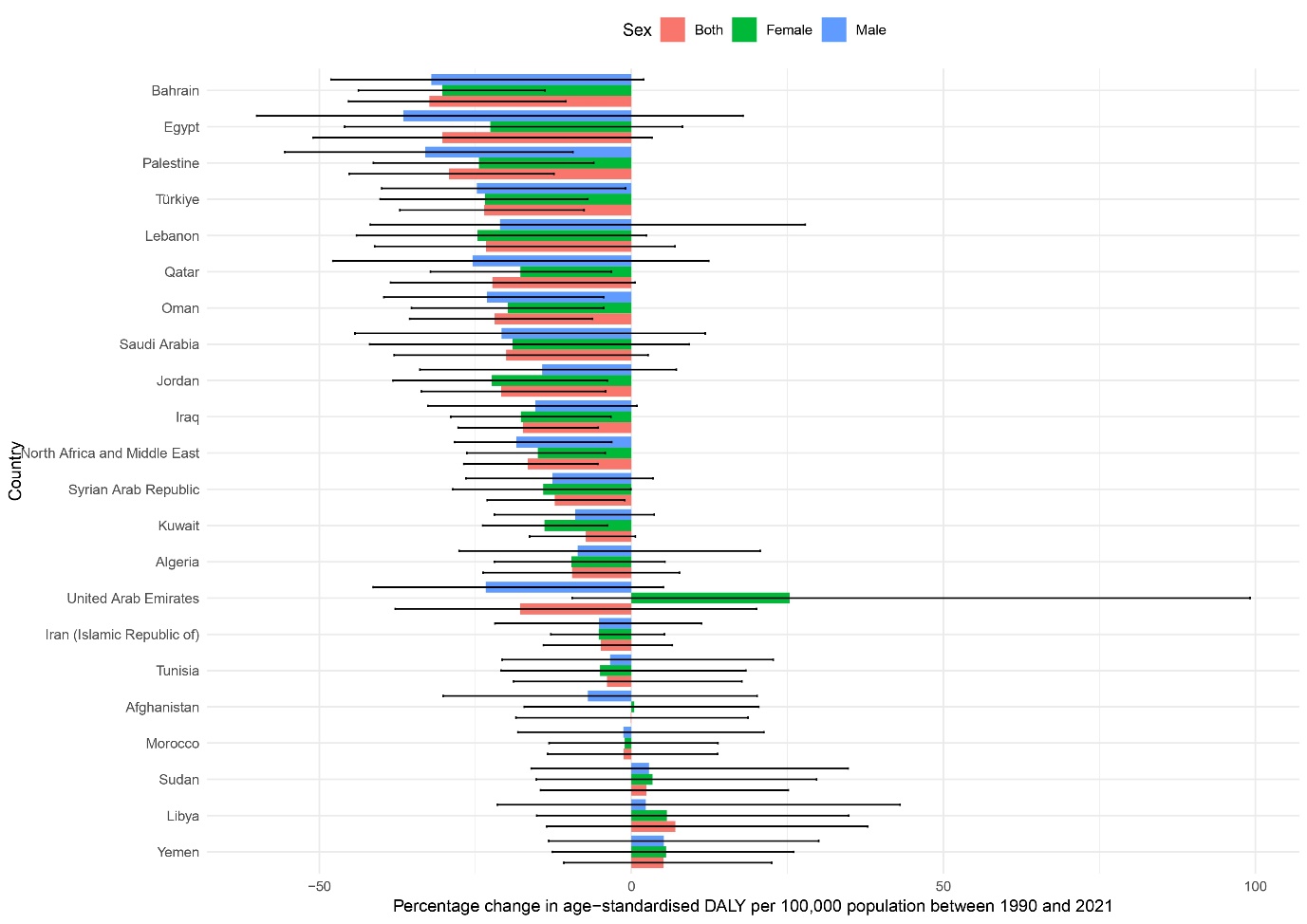
**

**Figure S3.** The percentage change in the age-standardized DALY per 100,000 population of gallbladder and biliary diseases in the Middle East and North Africa region from 1990 to 2021, by sex and country. DALY = disability-adjusted-life-year.
